# The effect of epilepsy on autistic symptom severity assessed by the social responsiveness scale in children with autism spectrum disorder

**DOI:** 10.1186/s12993-016-0105-0

**Published:** 2016-06-27

**Authors:** Chanyoung Ko, Namwook Kim, Eunjoo Kim, Dong Ho Song, Keun-Ah Cheon

**Affiliations:** College of Medicine, Yonsei University, 50 Yonsei-ro, Seodaemun-Gu, Seoul, 120-752 South Korea; Division of Child and Adolescent Psychiatry, Department of Psychiatry, College of Medicine, Severance Hospital, Yonsei University, Seoul, South Korea; Institute of Behavioral Science in Medicine, College of Medicine, Yonsei University, 50 Yonsei-ro, Seodaemun-Gu, Seoul, 120-752 South Korea; Department of Psychiatry, Institute of Behavioral Science in Medicine, College of Medicine, Gangnam Severance Hospital, Yonsei University, 211 Eonju-ro, Gangnam-gu, Seoul, 06273 South Korea

**Keywords:** Autism spectrum disorder, Epilepsy, Autistic symptom severity, Social responsiveness scale

## Abstract

**Background:**

As the prevalence of autism spectrum disorders in people with epilepsy ranges from 15 to 47 % (Clarke et al. in Epilepsia 46:1970–1977, 2005), it is speculated that there is a special relationship between the two disorders, yet there has been a lack of systematic studies comparing the behavioral phenotype between autistic individuals and autistic individuals with epilepsy. This study aims to investigate how the co-occurrence of epilepsy and Autism Spectrum Disorder (ASD) affects autistic characteristics assessed by the Social Responsiveness Scale (SRS), which has been used as a measure of autism symptoms in previous studies. In this research we referred to all individuals with Autism or Autistic Disorder as individuals with ASD.

**Methods:**

We reviewed the complete medical records of 182 participants who presented to a single tertiary care referral center from January 1, 2013 to July 28, 2015, and subsequently received complete child and adolescent psychiatric assessments. Of the 182 participants, 22 were diagnosed with Autism Spectrum Disorder and epilepsy. Types of epilepsy observed in these individuals included complex partial seizure, generalized tonic–clonic seizure, or infantile spasm. Using ‘Propensity Score Matching’ we selected 44 children, diagnosed with only Autism Spectrum Disorder, whose age, gender, and intelligence quotient (IQ) were closely matched with the 22 children diagnosed with Autism Spectrum Disorder and epilepsy. Social functioning of participants was assessed by the social responsiveness scale, which consists of five categories: social awareness, social cognition, social communication, social motivation, and autistic mannerisms. Bivariate analyses were conducted to compare the ASD participants with epilepsy group with the ASD-only group on demographic and clinical characteristics. Chi square and t test p values were calculated when appropriate.

**Results:**

There was no significant difference in age (p = 0.172), gender (p > 0.999), IQ (FSIQ, p = 0.139; VIQ, p = 0.114; PIQ, p = 0.295) between the two groups. ASD participants with epilepsy were significantly more impaired than ASD participants on some measures of social functioning such as social awareness (p = 0.03) and social communication (p = 0.027). ASD participants with epilepsy also scored significantly higher on total SRS t-score than ASD participants (p = 0.023).

**Conclusions:**

Understanding the relationship between ASD and epilepsy is critical for appropriate management (e.g. social skills training, seizure control) of ASD participants with co-occurring epilepsy. Results of this study suggest that mechanisms involved in producing epilepsy may play a role in producing or augmenting autistic features such as poor social functioning. Prospective study with larger sample sizes is warranted to further explore this association.

## Background

Autism spectrum disorder (ASD) is a childhood developmental disorder described by two core symptom dimensions—social communication and restricted, repetitive behavior (RRB) [2]. ASD encompasses a highly heterogeneous set of individuals with wide variations in clinical presentation, symptom severity, and cognitive ability [2]. Epilepsy is characterized by an enduring tendency to produce epileptic seizures and practically defined as having two un-triggered seizures occurring at least 24 h apart [3]. The co-occurrence of ASD and epilepsy is well recognized and has interested clinicians and researchers, yet the relationship between the two conditions has not been well established on a pathophysiologic level [4]. Prevalence estimates may vary, but between 11 and 39 % of individuals with ASD have been reported to develop epilepsy [4–6], which exceeds that of the general population (0.7–1 %) [7]. The prevalence of autism spectrum disorders in people with epilepsy ranges from 15 to 47 % [1].

Characteristics of individuals with both ASD and epilepsy have been explored in a handful of cross-sectional population-based studies [8–13]. Previous publications have reported findings about demographical variables such as age of onset, gender ratio, type of epilepsy, cognitive ability, and verbal ability. To date only one variable—lower cognitive ability—has consistently shown independent association with co-occurrence of epilepsy in individuals with ASD across all studies [8–13]. No specific epilepsy syndrome or seizure type has been associated, although focal or localization-related seizures are often reported [8]. While epilepsy onset in individuals without autism has been described to be highest in the first year of life [14, 15], in individuals with ASD, two peaks of seizure onset have been consistently reported, one in early childhood [16] and one in adolescence and continuing through adulthood [9, 17, 18]. Long-term follow-up cohort studies have shown higher prevalence of epilepsy in children with ASD of older age [8, 9, 13].

Only a few published studies have compared the clinical profiles of individuals who have both ASD and epilepsy with individuals who have only ASD [19–23]. Furthermore, little is known about the influence of epilepsy on the autistic symptoms in individuals with ASD. Clinical assessment of individuals with ASD and co-morbid epilepsy will give us an insight into how comorbid epilepsy affects the clinical features and natural history, certain cognitive-behavioral as well as psycho-pharmacological challenges associated with co-occurrence of ASD and epilepsy. One study showed a general trend towards greater developmental difficulties and stereotyped behaviors in children with epilepsy and ASD; their findings suggested that the presence of epilepsy may affect social functioning and incite behavioral problems [20]. Tuchman and Cuccaro found an association between epilepsy and autistic mannerisms such as repetitive object use and unusual sensory interests [17]. These studies provided the initial evidence suggesting that individuals with ASD and co-morbid epilepsy have elevated autism symptoms. One way of determining the role of epilepsy in autistic characteristics is through the use of quantitative assessment that is known to measure ASD symptom severity.

The social responsiveness scale (SRS), developed by Constantino et al. is a brief screening questionnaire completed by a third party informant that is often used to evaluate ASD symptom severity [24]. Although the SRS refers to a measure of social deficiency, many SRS items describe other core features of ASD such as autistic mannerisms [24]. The SRS provides an overall quantitative score as well as treatment specific sub-scores pertaining to receptive, cognitive, expressive, and motivational aspects of social behavior. Recent studies evaluating the efficacy of the SRS have shown that the SRS scores are influenced by age, gender, IQ, and presence of psychiatric co-morbidities [25]. Therefore, results from trials that employ this metric must be interpreted in light of these possible confounding factors. For the purpose of comparison studies or large cohort studies, screening tools such as SRS may be more appropriate and practical than standard ASD diagnostic tools, which can take several hours to complete [24]. This scale permits rapid detection, hence early diagnosis, of ASD while providing a good index of the severity of autistic social impairment [26].

Two previous studies have employed the SRS to ascertain the relationship between epilepsy and autism symptoms [19, 27]. Viscidi et al. showed that ASD children with epilepsy had more severe autism symptoms than ASD children without epilepsy, which was mostly explained by the lower IQ of the epilepsy group. After statistically adjusting for the effect of IQ, SRS scores of children with and without epilepsy did not differ significantly [19]. Wakeford et al. utilized an abbreviated version of the SRS, social responsiveness scale—shortened (SRS-S), to study autistics characteristics in adults with epilepsy. They found that higher SRS-S scores were associated with having diagnosis of epilepsy and were perceived to increase during seizure activity [27].

To our knowledge, only two published studies have investigated the relationship between autism and epilepsy using the SRS. Whereas Wakeford et al. suggested that seizure activity itself might have an impact on social difficulties, Viscidi et al. implied that diagnosis of epilepsy might be associated with social impairment solely due to the effect of cognitive impairment. Based on these studies, it is unclear whether seizure activities or having diagnosis of epilepsy has a direct impact on autism symptoms. Therefore, the current study aimed to ascertain this possibility by utilizing the SRS, to observe any difference between individuals with ASD and co-morbid epilepsy and matched control sample (individuals with only ASD) that have similar distributions on covariates such as age, gender, and IQ. We hypothesized that in the group of individuals diagnosed with both disorders, there would be an increase of ASD characteristics represented by higher SRS scores.

## Methods

### Patients and controls

**Fig. 1:**
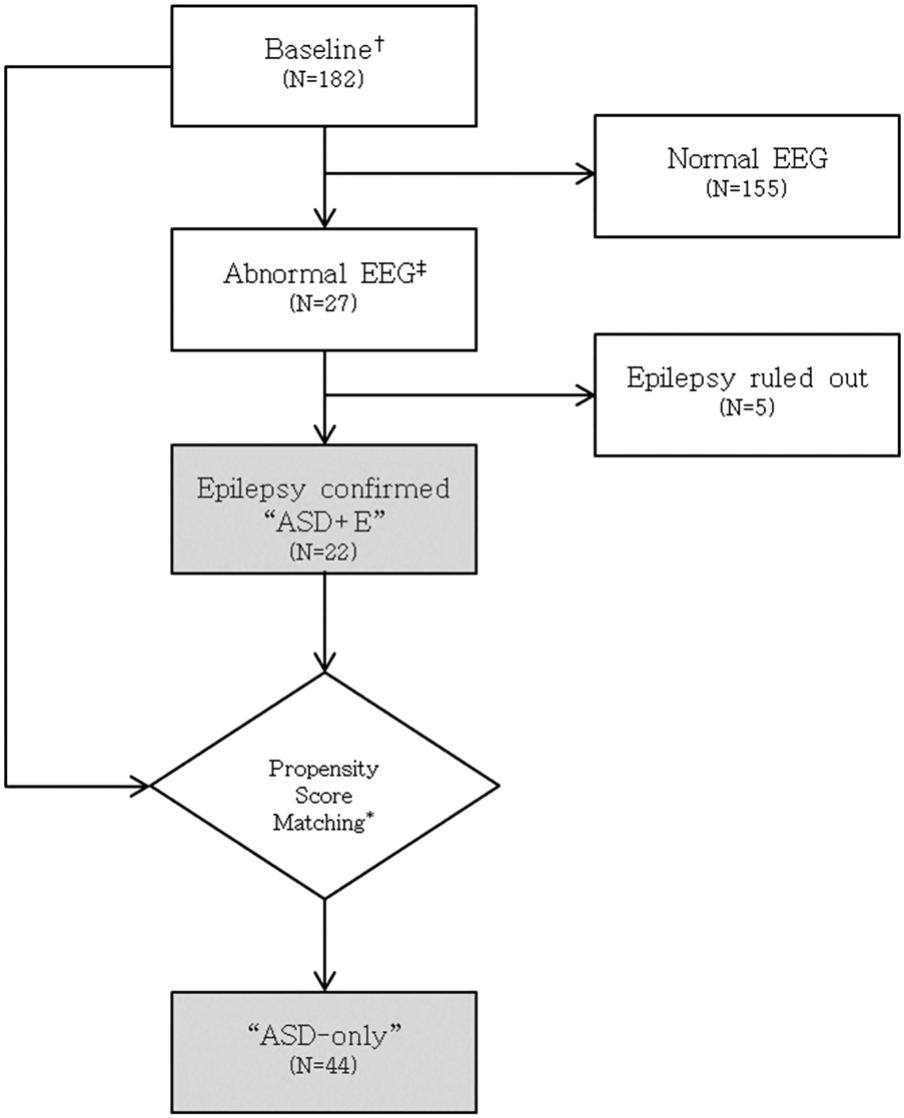
Flow chart describing the selection process of ASD + E and ASD-only groups. *Dagger* Participants enrolled in 2013–2015 who had completed the SRS interview and cognitive assessments. *Double dagger* Epileptiform discharges. *Asterisk* 1:2 ratio *propensity score matching* was conducted accounting for age, gender, and IQ. *ASD* *+* *E* ASD participants with epilepsy

All participants were originally seen at a specialist out-patient clinic for children with autism at Severance Children’s Hospital between January 1, 2013 and July 28, 2015. During this period, 182 patients had completed the SRS interview and routine developmental and cognitive assessments. Twenty seven of the 182 patients were reported to have shown epileptiform discharges on routine electroencephalogram (EEG); 22 of these 27 patients had been diagnosed with ASD and epilepsy (ASD + E). Forty-four ASD-only patients were selected from 155 patients with normal EEG using a statistical maneuver called *propensity score matching* [28]. *Propensity score matching* refers to a set of multivariate methods that estimate the effect of one factor by accounting for covariates known to affect the overall outcome [29]. This method allows the investigator to design and analyze an observational study mimicking certain characteristics of a randomized controlled trial [30]. For instance, conditional on the ‘propensity score,’ the distribution of observed baseline covariates will be similar between the participant and control group. A previous publication indicated that age, gender, and IQ influence scores on the SRS [25]. Accounting for these three covariates, one-to-two matching was performed, yielding 44 age-, gender-, and IQ- matched control ASD-only patients (Fig. 1). All study procedures were approved by the institutional review board at Severance Hospital, Yonsei University College of Medicine in Seoul, South Korea.

### Clinical assessment

All study participants had had a previous clinical diagnosis of ASD. Diagnoses of childhood autism or atypical autism were established using the Childhood Autism Rating Scale (CARS), a behavior rating scale intended to help diagnose autism [31], or autism diagnostic interview-revised (ADI-R), the “gold” standard for ASD diagnosis [32]. Epilepsy had been diagnosed previously by a pediatric neurologist. For the purposes of this study, epilepsy was defined as ‘two or more non-febrile seizures that were not confined to pre-school period (up to 5 years of age)’. The classification of seizure type followed the definitions of the International League against Epilepsy [3]. Individuals with neonatal seizures (i.e. seizures appearing before the age of 1 month which did not persist) were excluded from this study.

The medical records of all participants were retrospectively reviewed for demographic information, past medical history, medication history, main seizure type, age of seizure onset, and number of anti-epileptic drugs (AEDs) currently prescribed. Full Scale IQ (FSIQ), verbal IQ (VIQ), performance IQ (PIQ) were assessed with the Korean version [33] of the Wechsler Intelligence Scale for Children III [34], or the Korean version [35] of the Wechsler Adult Intelligence Scale III [36].

The SRS is a 65-item questionnaire that serves as a screening tool for ASD as well as a quantitative measure of ASD symptom severity in children aged 4 through 18. This scale was designed to be completed by an adult (parent or teacher) who is familiar with the child’s current behavior and developmental history [37]. In this study, we used SRS scores based on the parent-completed questionnaire. The SRS assesses five domains, which include: social awareness, social information processing, expressive social communication, social anxiety/avoidance, and autistic preoccupations/mannerisms. Each item is scored on a scale ranging from 1 (not true) to 4 (almost always true). Raw scores are converted to T-scores (with mean of 50 and standard deviation of 10) for gender and rater type. T-score of ≥76 is considered severe and strongly associated with a clinical diagnosis of ASD. T-score of 60-75 falls in the mild to moderate range and considered typical for high functioning ASD, while a T-score of ≤59 suggests an absence of ASD symptoms [37]. The internal consistency of the SRS with a Cronbach’s α >0.90 is considered high [24]. The SRS also has good inter-rater reliability of r = 0.91 [24, 38]. Studies have shown the SRS is capable of distinguishing individuals with pervasive developmental disorders such as ASD and other psychiatric disorders such as ADHD [24, 37]. Moderately strong associations were found between the SRS and the ADI-R, with correlation coefficients ≥0.52 across all subscales [24]. The SRS was translated into Korean language by Korean autism researchers and the Korean version of the SRS was back-translated into English by a bilingual child psychiatrist. The back-translated version was reconfirmed by a child and adolescent psychiatrist at the University of California San Francisco. Currently, the Korean version of the SRS has been well standardized and widely used (Cheon et al. under revision).

### Statistical analysis

Statistical analyses were performed using Statistical Package for Social Sciences (SPSS PC, version 20.0). Statistical significance was defined at a level of *p* < 0.05, and a p < 0.10 was regarded as a statistical trend toward change. Bivariate analyses were conducted to compare ASD participants with epilepsy group with the ASD-only group on demographic and clinical characteristics. Chi square and t test p values were calculated when appropriate.

## Results

### Participant characteristics

The characteristics of all individuals in the study are summarized in Table 1. The ASD participants with epilepsy group had been matched with comparison group (ASD-only) based on age, gender, and IQ. Consequently there was no statistical difference in age (p = 0.172), gender (p > 0.999), IQ (FSIQ, p = 0.139; VIQ, p = 0.114; PIQ, p = 0.295) between the ASD participants with epilepsy group and the ASD-only group. There was no statistically significant difference in gestational age (p = 0.386), birth weight (p = 0.072), obstetric complications (p = 0.485), use of antipsychotic medication (p = 0.191) between the two groups.

**Table 1:** Demographic characteristics of ASD + E and ASD-only participants

	ASD-only (N = 44)	ASD + E (N = 22)	p value
Age^a^ (years)	8.273 ± 4.326	10.227 ± 5.814	0.172
Gender^b^			
Male	43 (97.7 %)	21 (95.5 %)	>0.999
Female	1 (2.3 %)	1 (4.5 %)	
FSIQ^a^	62.318 ± 17.095	55.455 ± 18.397	0.139
VIQ^a^	67.80 ± 22.130	58.68 ± 21.007	0.114
PIQ^a^	62.73 ± 15.813	58.27 ± 16.799	0.295
Intellectual disability based on FSIQ^b^			
Non-intellectual disability IQ >70	12 (27.3 %)	5 (22.7 %)	0.102
Mild intellectual disability IQ 50–70	20 (45.5 %)	6 (27.3 %)	
Moderate-severe intellectual disability IQ <50	12 (27.3 %)	11 (50.0 %)	
Gestation age (weeks)^b^			
≤31	1 (2.3 %)	0 (0 %)	0.386
32–36	8 (18.2 %)	2 (9.1 %)	
37–41	35 (79.5 %)	54 (81.8 %)	
≥42	0 (0 %)	1 (4.5 %)	
Birth weight (g)^b^			
1500–2499	2 (4.5 %)	1 (4.5 %)	0.072
2500–3999	41 (93.2 %)	17 (77.3 %)	
4000–4499	1 (2.3 %)	4 (18.2 %)	
Obstetrics complication^b^			
No	38 (86.4 %)	17 (77.3 %)	0.485
Yes	6 (13.6 %)	5 (22.7 %)	
Antipsychotic medication^b^			
No	25 (56.8 %)	8 (36.4 %)	0.191
Yes	19 (43.2 %)	14 (63.6 %)	

*ASD* *+* *E* ASD participants with epilepsy

^a^Independent two sample t test

^b^Chi square test

### Co-morbid conditions

Within the ASD participants with epilepsy group, 4 (18.2 %) reported to have ADHD; 2 (9.1 %) reported to have Tuberous sclerosis, 2 (9.1 %) reported to have depression; 1 (4.5 %) reported to have bipolar disorder; 3 (13.6 %) reported to have Tourette syndrome; 5 (22.7 %) reported to have additional diagnoses (Table 2). Within the ASD-only group, 16 (36.4 %) reported to have ADHD; 2 (4.5 %) reported to have depression; 2 (4.5 %) reported to have psychosis; 5 (11.4 %) reported to have bipolar disorder; 3 (6.8 %) reported to have anxiety disorder; 2 (4.5 %) reported to have additional diagnoses (Table 2). Additional diagnoses included neurofibromatosis type 1, fragile-X syndrome, subarachnoid hemorrhage, organic brain syndrome, cortical dysplasia, and childhood onset parkinsonism. ASD participants with epilepsy group and ASD-only group showed statistical difference in the percentage of Tourette syndrome (p = 0.034) and additional diagnoses (p = 0.036) (Table 2).

**Table 2:** Reported co-morbid conditions in ASD + E and ASD-only participants

	ASD-only (N = 44) (%)	ASD + E (N = 22) (%)	p value
Attention deficit hyperactivity disorder (ADHD)			
No	28 (63.6)	18 (81.8)	0.163
Yes	16 (36.4)	4 (18.2)	
Tuberous sclerosis			
No	44 (100)	20 (90.9)	0.108
Yes	0 (0)	2 (9.1)	
Depression			
No	42 (95.5)	20 (90.9)	0.596
Yes	2 (4.5)	2 (9.1)	
Psychosis			
No	42 (95.5)	22 (100)	0.549
Yes	2 (4.5)	0 (0)	
Bipolar disorder			
No	39 (88.6)	21 (95.5)	0.655
Yes	5 (11.4)	1 (4.5)	
Anxiety disorder			
No	41 (93.2)	22 (100)	0.545
Yes	3 (6.8)	0 (0)	
Tourette disorder*			
No	44 (100)	19 (86.4)	0.034
Yes	0 (0)	3 (13.6)	
Other diagnoses^a,^*			
No	42 (95.5)	17 (77.3)	0.036
Yes	2 (4.5)	5 (22.7)	

*ASD* *+* *E* ASD participants with epilepsy

^a^Other diagnoses: neurofibromatosis-type 1, fragile-x, subarachnoid hemorrhage, organic brain syndrome, cortical dysplasia, childhood-onset parkinsonism

* p < 0.05

### Epilepsy profile of ASD participants with co-occurring epilepsy

Epilepsy variables of the ASD participants with epilepsy (N = 22) are summarized in Table 3. The mean age at onset of confirmed epilepsy was 5.57 years (SD = 4.71). The number of AEDs prescribed at time of assessment were 1.45 (SD = 1.10), which means that the majority of them were receiving one or two anticonvulsants. Of the 22 participants with co-morbid epilepsy, two were diagnosed with infantile spasms, 14 reported to have complex partial seizures, and six reported to have generalized tonic–clonic seizures (Table 3).

**Table 3:** Epilepsy variables (seizure onset age, number of AEDs, type of epilepsy)

	ASD-only (N = 44)	ASD + E (N = 22)
Age at seizure onset, years	–	5.57 ± 4.71
Number of current AEDs	–	1.45 ± 1.10
Infantile spasms	–	2 (3.0 %)
Complex partial seizures	–	14 (21.2 %)
Generalized tonic–clonic seizures	–	6 (9.1 %)

*AEDs* anti-epileptic drugs; *ASD* *+* *E* ASD participants with epilepsy

### Autistic symptom severity

Independent two sample t test was employed to compare the two samples in terms of their SRS ratings (Table 4). ASD participants with epilepsy scored generally higher than ASD-only participants across all SRS categories as represented by significantly higher SRS total t-score (p = 0.023). ASD participants with epilepsy showed significantly more marked severity in social awareness (p = 0.03) and social communication (p = 0.027). There was no statistical difference in social cognition (p = 0.081), social motivation (p = 0.0505), and autistic mannerisms (p = 0.065). However, the subscale scores for these three categories suggested a trend towards participants with ASD participants with epilepsy having greater severity in all three categories (p < 0.1).

**Table 4:** Mean differences between groups on SRS total and subscale scores

	ASD-only (N = 44)	ASD + E (N = 22)	p value
Total**	82.14 ± 17.323	92.41 ± 16.141	0.023
Social awareness**	63.84 ± 15.749	73.14 ± 16.485	0.03
Social cognition*	71.98 ± 14.387	78.41 ± 12.812	0.081
Social communication**	82.89 ± 18.975	93.36 ± 15.041	0.027
Social motivation*	76.64 ± 19.569	86.18 ± 15.522	0.0505
Autistic mannerisms*	84.36 ± 17.062	93.68 ± 22.474	0.065

Independent two sample t test

*ASD* *+* *E* ASD participants with epilepsy

* p < 0.1

** p < 0.05

## Discussion

Among children diagnosed with ASD, we found significant difference in autistic characteristics between children with and without epilepsy. Even after adjusting for baseline characteristics such as age, gender, and full scale IQ, ASD participants with epilepsy were found to be associated with higher scores on the SRS *total t*-*score*, *social awareness*, and *social communication*, indicating greater impairment. Based on our statistical model, there seemed to be a significant relationship between epilepsy and autistic characteristics in ASD children that is not explained by the association between epilepsy and low IQ. Participants diagnosed with both ASD and epilepsy appeared to be more socially impaired, especially in their capacity to pick up on social cues and organize expressive acts of social communication. In addition, ASD participants with epilepsy generally scored higher on other items that ascertain *social cognition* and *social motivation*.

Several studies have published in-depth reviews on the relationship between ASD and epilepsy [5, 8–10]. They have examined demographical variables such as the age of seizure onset, gender ratio, type of epilepsy, and intelligence level. Turk et al. was one of the first studies to compare the clinical profiles of matched groups of children with only ASD and children who were diagnosed with both ASD and epilepsy [20]. Utilizing the diagnostic interview for social and communication disorders (DISCO-11), they demonstrated that ASD participants with epilepsy were associated with greater motor difficulties, developmental delays, and challenging behavior in public places. Smith et al. study showed that individuals with intellectual disability (ID) combined with ASD and epilepsy were significantly more impaired than ID groups with a single co-morbid factor (ASD or epilepsy) on some measures of behavior problems including self-injury and disruptive behavior [21, 22]. No significant differences were found on stereotyped behaviors among all groups (ID-only vs. ASD-only vs. ASD and epilepsy vs. ID with ASD and epilepsy) [21]. Individuals with ID expressing co-morbid ASD and epilepsy had significantly more impaired social skills (e.g. sharing interests, playing, smiling, and communicating using gestures) than groups containing a single factor (ID, ASD, or epilepsy only) [22]. Viscidi et al. underscored the large effect of IQ on the relationship between epilepsy and ASD as they failed to find a relationship between epilepsy and more severe autism symptoms after adjusting for IQ [19].

Findings from Smith et al. and Viscidi et al., therefore, implied that ASD children with epilepsy are at risk of having more severe autism symptoms due to the increased chance of these children having lower IQ. It is well established that cognitive impairment is an independent risk factor for developing epilepsy in individuals with ASD [12]. However, even the low rates of epilepsy reported in individuals with ASD without intellectual disability is higher than the general population rate; therefore, there is an increased risk of epilepsy in ASD even in the absence of intellectual disability [12]. In order to examine the effect of epilepsy on autistic symptom severity, without the influence of IQ, we designed a study that matched the ‘ASD-only participants’ with ‘ASD participants with epilepsy’ based on IQ measurements. As a result, there were no significant differences in FSIQ, VIQ, and PIQ between the two groups; consequently, any difference in SRS scores between the two groups would be due to the effect of epilepsy rather than lower cognitive ability.

Commensurate with previous studies [17, 20], our data indicated that individuals with ASD and epilepsy are more likely to be reported as having *autistic mannerisms*; however, statistical significance was not reached (p = 0.065). Our current data lack the statistical power to support the hypothesis that individuals with ASD and epilepsy are significantly more impaired than ASD-only participants on measures of RRBs; thus, it is not certain whether co-occurrence of epilepsy affects the development of autistic mannerisms. In previous studies, RRBs did not correlate with social communicatory difficulties in individuals with ASD, suggesting dissociation between the two symptom domains [39, 40]. One plausible hypothesis is that epilepsy plays a role in social functioning while having no effect on stereotypical behavior. Future direction for this research is to verify any correlation between RRBs and social-communicatory difficulties in ASD participants with epilepsy.

It is well established that epilepsy is more prevalent among individuals diagnosed with ASD than in the normal population [12, 15]. The vice versa is true as well [1]. However, very little do we know about the traits and characteristics of individuals with ASD and epilepsy and the common mechanisms linking the two types of disorders. While previous studies have highlighted the high co-morbidity between epilepsy and autism, there has been a lack of detailed examination of how certain hallmark features of autism such as impaired social functioning may be present in heterogeneous groups of individuals with epilepsy. One explanation might be that poor social skills are missed at the diagnostic-clinical level during assessment for epilepsy. Furthermore, studies of social cognition in epilepsy have been neglected, partly owing to findings from recent studies, which demonstrated a lack of association between epilepsy and social functioning after accounting for differences in IQ [18]. On the other hand, some studies have indicated that epilepsy can affect brain structures and neural networks associated with social cognition [41]; such findings allude to the possibility that pathogenesis of epilepsy may affect social functioning. Furthermore, social cognitive abilities in children may be associated with seizure frequency [42, 43]. To date, several studies attempted to establish the association between epilepsy and social cognitive abilities [19, 20, 23, 27, 44].

In contrast to findings from the Viscidi et al. study [19], our results demonstrated that there is a significant association between the two neurological conditions irrespective of difference in IQ. However, no further conclusion can be drawn in regard to whether co-occurrence of epilepsy causes elevated autism symptoms and disrupt social cognitive abilities since we do not understand the extent to which social functioning is shaped by neurobiological and psychological factors.

Nevertheless, findings of the present study point to several important clinical implications. First, individuals with ASD and epilepsy are more likely to have severe social impairments than those diagnosed solely with ASD. Secondly, individuals with ASD and epilepsy would benefit from an intensive social skills training, and aggressive treatment approaches for epilepsy may prevent decline in social functioning. Thirdly, the incidence of epilepsy may be higher for individuals with ASD who scored higher on the SRS; these individuals may benefit from thorough neurologic assessments and evaluation for epilepsy as part of their routine follow-up.

### Limitations and strengths

A few limitations should be considered when interpreting the results of the current study. First, sample sizes of this study were relatively small; replication of the current findings with a larger sample is warranted. A large-sample birth cohort study as well as a study assessing the severity of autistic symptoms in relation to onset of epilepsy may further elucidate the cause-and-effect relationship between epilepsy and ASD. Second, much of the information was retrospectively gathered and based on parent report, so may have been participant to recall and other biases. Third, the study sample was selected from a clinic population following exclusion of individuals without a complete SRS measurement, which suggests exclusion of individuals with very severe intellectual disabilities who were unable to complete the interview. Thus, ASD with epilepsy sample included in this study is not necessarily representative of all children with epilepsy and autism. Fourth, *propensity score matching* is the observational study analog of randomization and that it can only balance the distribution of observed covariates, whereas randomization balances the distribution of observed as well as unobserved covariates. Fifth, despite successfully achieving group-matching using *propensity score matching*, the ASD-only group appeared to have a slight trend for higher FSIQ (p = 0.102). Although not statistically significant, ASD-only group may appear to have less intellectual disability than the ASD participants with epilepsy group. Sixth, although small in number, ASD participants with epilepsy group had more additional diagnoses such as neurofibromatosis type 1, fragile-x, organic brain syndrome, etc., which may also explain the significant difference in social functioning between the two groups. We were not able to control for these co-morbid conditions using the propensity score matching; hence, impaired social functioning in the ASD participants with epilepsy may not be a function of epilepsy alone. In spite of these limitations, there are also some positive aspects. Strengths of this study lie in the utility of the SRS as a measure of autistic symptom severity and the methodology in selecting a well-matched control group. Using the *propensity score matching*, we were able to construct ASD participants with epilepsy and matched control samples (ASD-only) that have similar distributions on covariates such as age, gender, and IQ. In contrast to Viscidi et al. study [19], which used the Poisson regression models and generalized linear models to adjust for the covariates in the later stages of data analysis, we employed a proven statistical technique that accounted for these covariates from the beginning. As a result, complicated multiple regression analyses and statistical errors associated with such analyses could be avoided. Previous studies on ASD were prone to pre-select those having DSM-IV autistic disorder with little reference to the frequently associated cognitive impairment or co-morbidities such as epilepsy. Careful statistical measures were taken to account for several variables that were previously reported to confound any analysis of population studies of autism.

## Conclusions

The co-occurrence of epilepsy and ASD is quite frequent and poses numerous challenges for the affected individuals including increased risk of worsened cognitive and behavioral profiles and overall worse prognosis. In the current study, individuals with ASD and co-morbid epilepsy appear to be at a higher risk for worsened social functioning. Large systematic studies employing strict ascertainment of samples, certain statistical tools to control for confounding factors such as IQ and other co-morbid conditions, as well as appropriate longitudinal follow-up are necessary to better shed light on the relationship between ASD and epilepsy. Early detection of social deficits as well as intensive social skills training should be considered as an integral part of their long-term care plans. Given that ASD and epilepsy affect one another’s behavioral phenotype as well as response to psychopharmacological treatment, proper management for epilepsy may in turn reduce autistic symptom severity in these individuals with ASD and epilepsy.

## Abbreviations

ASD: autism spectrum disorder
RRB: restricted, repetitive behavior
SRS: social responsiveness scale
IQ: intelligence quotient
FSIQ: full scale intelligence quotient
VIQ: verbal intelligence quotient
PIQ: performance intelligence quotient
AED: antiepileptic drug
EEG: electroencephalogram
ASD + E: ASD participants with epilepsy
CARS: childhood autism rating scale
ADI-R: autism diagnostic interview-revised
SPSS: statistical package for social sciences
ADHD: attention deficit hyperactivity disorder
